# Effects of co-formulants on the absorption and secretion of active substances in plant protection products* in vitro*

**DOI:** 10.1007/s00204-021-03140-x

**Published:** 2021-08-20

**Authors:** Mawien Karaca, Benjamin Christian Fischer, Christian Tobias Willenbockel, Tewes Tralau, Philip Marx-Stoelting, Denise Bloch

**Affiliations:** 1 Department of Pesticides Safety, German Federal Institute for Risk Assessment, Max-Dohrn-Straße 8-10, 10589 Berlin, Germany; 2 Institute for Chemistry, Technical University of Berlin, Straße des 17. Juni 115, 10623 Berlin, Germany

**Keywords:** Mixture effects, Co-formulants, Surfactants, Toxicokinetic, Plant protection products, Pesticides

## Abstract

Currently, the authorisation process for plant protection products (PPPs) relies on the testing of acute and topological toxicity only. Contrastingly, the evaluation of active substances includes a more comprehensive set of toxicity studies. Nevertheless, mixture effects of active ingredients and co-formulants may result in increased toxicity. Therefore, we investigated effects of surface active co-formulants on the toxicity of two PPPs focussing on qualitative and quantitative toxicokinetic effects on absorption and secretion. The respective products are based on the active substances abamectin and fluroxypyr-meptyl and were tested for cytotoxicity in the presence or absence of the corresponding surfactants and co-formulants using Caco-2 cells. In addition, the effect of co-formulants on increased cellular permeation was quantified using LC–MS/MS, while potential kinetic mixture effects were addressed by fluorescence anisotropy measurements and ATPase assays. The results show that surface active co-formulants significantly increase the cytotoxicity of the investigated PPPs, leading to more than additive mixture effects. Moreover, analytical investigations show higher efflux ratios of both active substances and the metabolite fluroxypyr upon combination with certain concentrations of the surfactants. The results further point to a significant and concentration-dependent inhibition of Pgp transporters by most of the surfactants as well as to increased membrane fluidity. Altogether, these findings strongly support the hypothesis that surfactants contribute to increased cytotoxicity of PPPs and do so by increasing the bioavailability of the respective active substances.

**Supplementary Information:**

The online version contains supplementary material available at 10.1007/s00204-021-03140-x.

## Introduction

Plant protection products (PPPs) are mixtures that, apart from the active substances, also contain a wide variety of co-formulants. Co-formulants provide PPPs with required properties for their application, thereby supporting the efficacy of active substances (Hazen 2000). In the context of PPP toxicity, it is usually the active substances that are assumed to be the main drivers of toxicity. Consequently, Regulation (EU) 283/2013 requires comprehensive mammalian toxicity testing for acute, chronic and sub-chronic effects only for the active substance but not for PPPs (EC 2013a). The latter are mainly evaluated for acute effects with their admission requiring tests for acute toxicity, irritation and skin sensitisation (EC 2013b). The co-formulants used therein do not require any further particular toxicological evaluation or authorisation as part of PPP Regulation (EU) 1107/2009 (EC 2009). Instead they are commonly subject to the REACH regulation and hence toxicologically tested and assessed depending on their annual production volume.

Although co-formulants are by definition non-active, they contribute to toxicodynamic or toxicokinetic mixture effects, potentially resulting in the altered toxicity of the PPP. In recent years several articles were published comparing active substances and PPPs, showing that the products may exhibit increased toxic effects (Adler-Flindt and Martin 2019; Hernandez et al. 2013; Zahn et al. 2018). Furthermore, Regulation (EU) 1107/2009 requires that “interaction between the active substance, safeners, synergists and co-formulants shall be taken into account” in the evaluation and authorisation of PPPs (EC 2009).

Yet, PPP risk assessment traditionally focuses on active substance(s) without particularly assessing specific effects of co-formulants (EC 2009). Instead, PPP hazard assessment is often based on the CLP calculation method, which relies on information on single substances and the additivity principle (EC 2008). Assuming a similar mode of action for the ingredients, this approach is viewed as a worst-case estimation of mixture effects (Backhaus et al. 2004; Junghans et al. 2006). It should be noted, however, that this assumption only applies to the toxicodynamic properties, and less so to potential toxicokinetic interactions (Van Cott et al. 2018). This in turn can lead to a potential underestimation of mixture effects as also indicated by previous studies (Van Cott et al. 2018). Also, Kurth et al. (2019) observed discrepancies between the hazard of PPPs classified in vivo and the results of the calculation method for acute oral and inhalation toxicity.

Therefore, there is a need for a more systematic approach to better understand the influence of co-formulants on the active substance’s toxicokinetics. Synergistic effects through toxicokinetic interactions may occur, for example by co-formulants significantly increasing the uptake or absorption of the active ingredient or other components leading to higher bioactivity or bioavailability (Kienzler et al. 2014).

Several chemicals with surface active properties may enhance the oral bioavailability of active substances/drugs as described previously (Weinheimer et al. 2017). The absorption enhancement can be due to increased membrane fluidity resulting in an increased passive transport flux (Woodcock et al. 1992) and/or due to the inhibition of active transporters (Li-Blatter et al. 2009). Furthermore, an interference with the tight junctions, especially for anionic surfactants, has been described (Aungst 1999).

Several surfactants, especially those with non-ionic properties, have been described to inhibit transporters in the gastrointestinal tract, i.e. tweens, cremophors and pluronic block copolymers (Nerurkar et al. 1996; Rege et al. 2001; Woodcock et al. 1992). Mostly the P-glycoproteins (Pgp), also known as multidrug resistance protein 1 (MDR1) or ABCB1, are effectively inhibited by these substances. Their further ability to alter membrane fluidity and thereby forcing a conformational change of Pgp is assumed to lead to the inhibition of Pgp’s ATPase activity (Dudeja et al. 1995; Woodcock et al. 1992). It has also been reported that Pgp inhibition may be induced by the polyoxyethylene (POE) structure of these surfactants, caused by changes in membrane fluidity (Hugger et al. 2002).

In the present paper, we focused on the impact of co-formulants with surface active properties on the absorption of active substances in PPPs. Our main interest was the investigation of effects as they may occur when handling PPPs. Two PPPs containing surface active co-formulants, used as emulsifiers, dispersing or wetting agents, were selected for further investigation: an insecticide containing abamectin and an herbicide containing fluroxypyr-meptyl.

The aim of this work was to investigate toxicokinetic mixture effects of surface active co-formulants and active substances on absorption and secretion in vitro. Transport studies were performed using Caco-2 cells, followed by quantification of the respective active ingredient using LC/MS–MS. Furthermore, concomitant fluorescence anisotropy measurements were conducted to assess potential effects on membrane fluidity. In addition, ATPase assays were applied to investigate the substances’ interaction with the active xenobiotic efflux transporter Pgp.

## Materials and methods

### Test compounds

Analytical grade abamectin (CAS no. 71751-41-2; batch no. BVBZ3693; purity 94.23%), analytical grade fluroxypyr-meptyl (CAS no. 81406-37-3; batch no. BCBT2272; purity 98.5%), analytical grade fluroxypyr (CAS no. 69377-81-7; batch no. BCCC5176; purity 99.1%) and Tween^®^ 80 (CAS no. 9005-65-6; batch no. S7769787925) were obtained from Sigma–Aldrich (Taufkirchen, Germany). Soprophor^®^ BSU (CAS no. 99734-09-5; batch no. 8361010), Soprophor^®^ 3D33 (CAS no. 90093–37-1; batch no. 8487267) and Rhodacal^®^ 60/BE (CAS no. 26264-06-2/104-76-7; batch no. 52573L) were purchased from Solvay AG (Brussels, Belgium). Emulsogen^®^ EL400 (CAS no. 61791-12-6; batch no. ESD0030925) was purchased from Clariant AG (Frankfurt am Main, Germany) and Solgad^®^ 150 ULN (CAS no. 64742-94-5; batch no. 0001201806) from OQEMA AG (Mönchengladbach, Germany). The PPPs were directly obtained from the manufacturer. Not all co-formulants contained in the PPPs were investigated individually, as their amounts in the products are negligible and/or they do not have any surface active properties. The composition information only on the investigated ingredients of the plant protection products are given in Table 1.

**Table 1:** Composition information on the investigated ingredients of the plant protection products

	Product 1	Product 2
Active substance	≈ 2% (w/w) abamectin CAS-Nr.: 71751-41-2 Containing ≥ 80% avermectin B_1a_ and ≤ 20% avermectin B_1b_	≈ 30% (w/w) fluroxypyr-meptyl CAS-Nr.: 81406-37-3
Investigated co-formulants	≈ 9% (w/w) Tween^®^ 80 CAS-Nr.: 9005-65-6 Polyoxyethylen-80-sorbitanmonooleat Non-ionic surfactant ≈ 2% (w/w) Soprophor^®^ BSU CAS-Nr.: 99734-09-05 Tristyrylphenol ethoxylate Non-ionic surfactant ≈ 2% (w/w) Soprophor^®^ 3D33 CAS-Nr.: 90093-37-1 Tristyrylphenol ethoxylate phosphate ester Non-ionic surfactant	≈ 7% (w/w) Emulsogen^®^ EL400 CAS-Nr.: 61791-12-6 Castor oil ethoxylate Non-ionic surfactant ≈ 4% (w/w) Rhodacal^®^ 60/BE CAS-Nr.: 26264-06-2/104-76-7 Calcium dodecylbenzene sulfonate Anionic surfactant ≈ 60% (w/w) Solgad^®^ 150 ULN CAS-Nr.: 64742-94-5 Solvent naphtha (petroleum), heavy aromatic (C9-C16) Organic solvent

All test compounds were dissolved in dimethyl sulfoxide (DMSO; CAS no. 67-68-5; batch no. 8O014081), purchased from Carl Roth (Karlsruhe, Germany), to result in a final solvent concentration of 0.4% (v/v) in cell culture medium.

### Cell culture

Caco-2 cells (ECACC: 86010202) were purchased from the European Collection of Authenticated Cell Cultures (Salisbury, UK). Caco-2 cells, passage 22-26, were grown and differentiated for three weeks in a culture medium, containing Dulbecco’s Modified Eagle Medium (DMEM; PAN-Biotech GmbH, Aidenbach, Germany), supplemented with 10% standardized fetal calf serum (FCS Superior; Capricorn Scientific GmbH, Ebsdorfergrund, Germany), 100 U/mL penicillin and 100 μg/mL streptomycin (PAA Laboratories GmbH, Pasching, Austria). The cells were cultivated at 37 °C, 5% CO_2_ and 5% humidity atmosphere in a cell culture incubator. They were passaged when reaching 80–90% confluence every 2 to 3 days. Passaging was performed at maximum ten times and by aspirating the medium, washing with phosphate-buffered saline (PBS) and incubating with trypsin–EDTA (0.05%) in Dulbecco’s phosphate-buffered saline (Capricorn Scientific GmbH, Ebsdorfergrund, Germany) at 37 °C for 3–5 min. The incubation was stopped by DMEM supplemented with FCS Superior and the cells were separated by centrifugation.

### Cell viability

Cytotoxicity was first analysed by water soluble tetrazolium assay (WST-1; Roche, Berlin, Germany), followed by neutral red uptake (NRU) assay. Caco-2 cells were seeded in 96-well plates at a density of 5000 cells per well and differentiated for three weeks. The cells were incubated with both active substances and all of the above-mentioned co-formulants individually, with a combination of the active substances and the respective surfactants in a ratio in accordance with the products, as well as with the respective products, containing all co-formulants, for 24 h. Eight different concentrations were investigated in culture medium with a final concentration of 0.4% DMSO. Triton X-100 (0.1%) served as a positive control. After 24 h incubation a WST-1 assay was performed according to the protocol provided by the supplier.

Cytotoxicity was analysed by NRU assay according to the protocol by Repetto et al. (2008). In brief, medium was replaced by 100 µL neutral red medium per well. Cells were incubated for 2 h at 37 °C and neutral red medium was removed. To extract the neutral red dye, 150 µL of a solution (50% ethanol/1% acetic acid) was added per well and the plates were shaken for 10 min at room temperature. Absorbed dye was quantified by fluorescence measurement (Excitation 530 nm; Emission 645 nm) on an Infinite M200 PRO plate reader (Tecan, Maennedorf, Swiss). Signals were background corrected and viabilities were expressed as percentage of untreated cells. Each sample was measured in six replicates. Three independent experiments were performed (*n* = 3) and means, as well as standard deviations (SD) were calculated.

### Concentration-additivity modelling

Dose–response modelling of concentration-additivity was used to examine the observed mixture effects, taking into account the cytotoxic effects of the investigated co-formulants. PROAST software ver. 70 was used to evaluate the nature of the observed mixture effects. Dose–response curves of the single compounds (active substances and co-formulants) were used to model a theoretical mixture curve based on the assumption of dose addition. Following the methods of Kienhuis et al. (2015), we fitted a four parameter exponential model: $y = a \times [c - (c - 1) \times \exp ( - b \times x^{d} )]$ to the single substances that showed cytotoxic effects (abamectin, Soprophor^®^ BSU and Soprophor^®^ 3D33 for product 1; fluroxypyr-meptyl, Rhodacal^®^ 60/BE and Solgad^®^ 150 ULN for product 2). Cytotoxicity dose–response data of mixtures and products were plotted in addition to the dose–response data of the respective single substances and their dose–response curve under the assumption of dose addition. The location of the plotted cytotoxicity dose–response data of the mixtures and the products were compared with the modelled dose–response curve under the assumption of dose addition (Kienhuis et al. 2015; Lasch et al. 2020).

### Fluorescence anisotropy

The impact of the surface active co-formulants Tween^®^ 80, Soprophor^®^ BSU, Soprophor^®^ 3D33, Rhodacal^®^ 60/BE and Emulsogen^®^ EL 400 and the solvent Solgad^®^ 150 ULN on the membrane fluidity of the Caco-2 cells were assessed by measuring DPH fluorescence anisotropy, according to a method described earlier with minor modifications (Zeng et al. 2012). Furthermore, the active ingredients and the products were investigated at sub-cytotoxic concentrations to rule out any impact of the active ingredients. Briefly, Caco-2 cells were cultivated in six-well plates for 21 days and detached on day 21 using 1 mL Accutase Cell Detachment Solution (ACC-1B, Capricorn Scientific GmbH, Ebsdorfergrund, Germany) per well. After incubation for 20–30 min at room temperature, detached cells were separated by centrifugation. Cells were resuspended in 2.5 mL of the test compounds or in 2.5 mL of the known membrane fluidizer benzyl alcohol (final concentration 60 mM; positive control) and incubated for 1 h at 37 °C. Afterwards Caco-2 cell suspensions (2 × 10^5^ cells / ml) were labelled by adding 5 µL of 1 mM 1,6-diphenyl-1,3,5-hexatriene (DPH) stock solution in tetrahydrofuran (THF) for 30 min at room temperature in darkness. Fluorescence anisotropy measurements were performed at 37 °C using a dual monochromator fluorescence spectrometer (model LS 55, PerkinElmer, Rodgau, Germany) fitted with polarizing filters and a stirred cell. Samples were excited with 355 nm vertical polarised light and emission was measured at 430 nm in vertical and horizontal direction. Since fluorescence anisotropy is inversely related to membrane fluidity, decreased DPH fluorescence anisotropy implies an increased membrane fluidity. Three independent experiments were performed (*n* = 3). The fluorescence anisotropy (*r*) is defined by following equation:

$$r = \left( {I_{VV} - GI_{VH} } \right)/\left( {I_{VV} + 2GI_{VH} } \right)$$

I_VV_: fluorescence intensity measured in direction parallel to the polarised exciting light.I_VH_: fluorescence intensity measured in direction perpendicular to the polarised exciting light.G: IVH/IHH; correction factor for the instrument.

### Pgp ATPase assay

This study focused on potential effects on Pgp transporters. Additional transporters present in Caco-2 cell lines may lead to potential cross-reactivity, the respective assay hence relied on recombinant human Pgp transporters integrated in membrane fractions.

The surface active co-formulants and the solvent Solgad^®^ 150 ULN were tested at sub-cytotoxic concentrations for Pgp inhibition after stimulation with verapamil. Abamectin was tested for Pgp inhibition with verapamil, as well as for substrate properties without verapamil, since abamectin has been described as a mammalian Pgp substrate with inhibitory properties at higher concentrations (Lespine et al. 2007). Furthermore, fluroxypyr-meptyl was tested for Pgp substrate properties. Pgp ATPase activity modulation was determined using the Pgp-Glo assay system (Promega, Madison, WI, USA) following the manufacturer's user protocol. Briefly, 25 µg of recombinant human Pgp membrane in Pgp-Glo assay buffer was incubated with different concentrations of each active substance or co-formulant in combination with 200 µM verapamil at 37 °C for 5 min. Furthermore, Pgp membrane was incubated with 100 µM Na_3_VO_4_ (selective inhibitor), 200 µM verapamil (substrate) or Pgp-Glo assay buffer (negative control). 5 mM MgATP was added to each well and 96-well plates were incubated at 37 °C for 40 min to initiate the reaction. Luminescence of the remaining, unmetabolised ATP was initiated by adding 50 µl of 25 mM ATP detection reagent. After briefly shaking on a plate shaker, 96-well plates were incubated at room temperature for 20 min. The luciferase generated luminescence signal was measured on an Infinite M200 PRO plate reader (Tecan, Maennedorf, Swiss). All measurements were corrected by subtraction of Na_3_VO_4_-treated signals (non Pgp ATPase activity) and represented as changes in luminescence signals (ΔRLU). Basal Pgp ATPase activity is represented by the difference in luminescence signals between Na_3_VO_4_-treated samples and untreated samples. Changes in luminescence (ΔRLU) caused by the co-formulants and abamectin incubated with verapamil were compared to ΔRLU caused by verapamil. ΔRLU values of the active ingredients (without verapamil) were compared to ΔRLU values of untreated samples (basal ΔRLU). Each condition was measured in four replicates (*n* = 4).

### Transport studies

Transport studies were conducted to investigate possible effects on the epithelial integrity caused by co-formulants, potentially leading to increased intestinal uptake of the active ingredients. Accordingly, quantification of abamectin and fluroxypyr-meptyl using LC–MS/MS analysis was conducted. Since in preliminary measurements very low amounts of fluroxypyr-meptyl were quantified, due to high metabolic conversion, fluroxypyr as one known metabolite has also been quantified. The acid fluroxypyr is formed by ester hydrolysis and has also herbicidal properties.

**Fig. 1:**
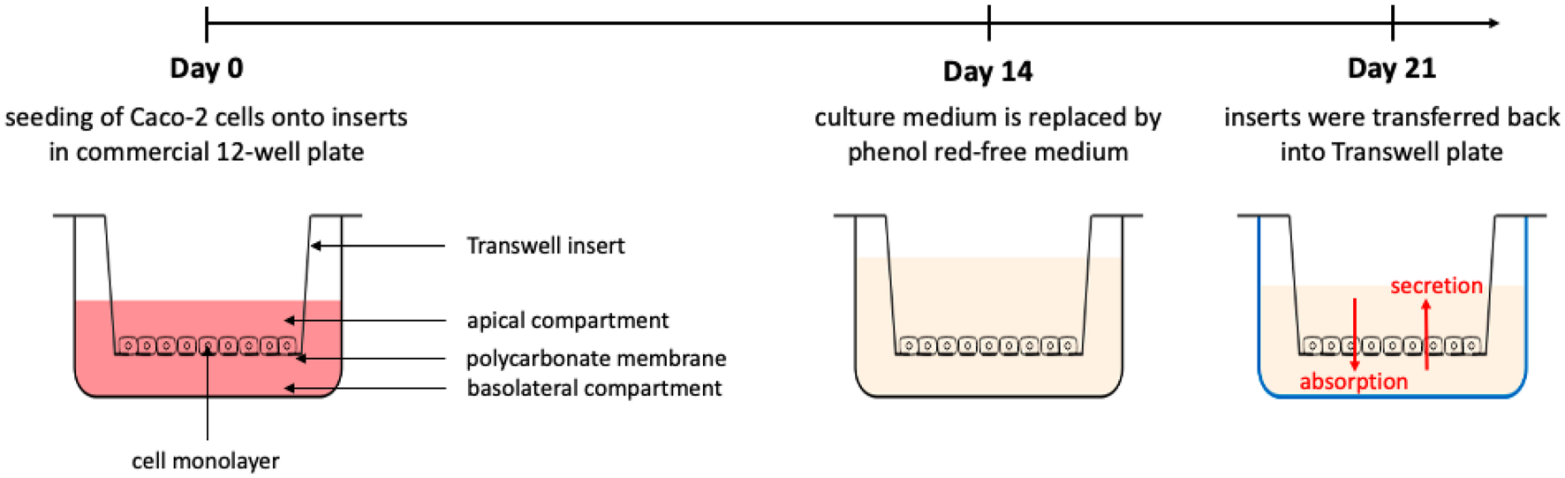
Overview of cultivation and experimental conditions of the transport studies

Transport studies of the active substances were performed as shown in Fig. 1 in 12-well transwell plates with inserts of polycarbonate membranes, 1.12 cm^2^ growth area and 0.4 μm pore size (Corning Incorporated, New York, USA). Caco-2 cells were seeded at a density of 50,000 cells onto inserts transferred into commercial 12-well plates and cultivated for 21 days at 37 °C, 5% CO_2_ and 5% humidity atmosphere as described earlier (Stock et al. 2019). Culture medium was changed every two to three days.

21 days after seeding and 18–24 h after medium change, inserts were transferred back into transwell plates. Transport studies were performed in apical (AP) to basolateral (BL) direction (absorption) and in BL to AP direction (secretion). Cells were exposed to (A) 1 mg/mL active substance individually, (B) 1 mg/mL active substance and the respective surfactants in a ratio corresponding to the products (mix 1A and mix 2A), (C) 1 mg/mL active substance and the respective surfactants in their highest individually sub-cytotoxic concentration (mix 1B and mix 2B) and (D) 1 mg/mL active substance in the respective product. Donor compartments were filled with 500 µL (AP) or 1500 µL (BL) of the compounds solved in phenol red-free culture medium, containing 0.4% DMSO. Receiver compartments were filled with 500 µL (AP) or 1500 µl (BL) phenol red-free culture medium containing 0.4% DMSO.

Cells were incubated at 37 °C for 8 h. Sample aliquots of 200 µL (AP) or 600 µL (BL) were taken from the receiver compartment at time intervals of 2, 4, 6 and 8 h. Sample aliquots were replaced with fresh, pre-warmed phenol red-free culture medium containing 0.4% DMSO, to ensure sink conditions. Sample aliquots were stored at − 80 °C and analysed by LC–MS/MS. Each transport study was performed in triplicate (*n* = 3) and means, as well as SD were calculated.

### Cell layer integrity

Cell layer integrity was monitored as a quality control by transepithelial electrical resistance (TEER) measurements and by fluorescein isothiocyanate-dextran (FITC-dextran) flux. A epithelial voltohmmeter with chopstick electrode (EVOM2, World Precision Instruments, Sarasota, USA) was used to measure TEER values as the electrical resistance between the AP and the BL side of the cell layer, as previously described (Lichtenstein et al. 2015). TEER measurements were performed at the beginning (0 h) and end (8 h) of each experiment. FITC-dextran flux was measured by adding 500 µL of 1 mg/mL 10 kDa FITC-dextran into the AP compartment. Its transport was measured after 22–24 h incubation at 37 °C in the BL compartment by fluorescence measurement (Excitation 485 nm; Emission 535 nm) on an Infinite M200 PRO plate reader (Tecan, Maennedorf, Swiss). Apparent permeability coefficients (Papp) were calculated by the formula: Papp = (ΔQ/Δt) × (A × c0)^−1^ for each well as described in detail earlier by Lichtenstein et al. (2015). TEER values were measured after incubation with FITC-dextran. A graphical visualisation of the cell layer integrity data can be found in the supplementary material (Fig. S2-3).

### Sample preparation

BL and AP medium samples were prepared with a Quick, Easy, Cheap, Effective, Rugged and Safe (QuEChERS) method first described by Anastassiades et al. (2007). Minor modifications were performed here. In brief, (1) 90% of the sample aliquots (180 µL or 540 µL) were added into 50 mL centrifuge tubes; (2) 5 mL of Mili-Q water were added; (3) 10 mL of acetonitrile (ACN) were added to abamectin containing samples/ 10 mL of ACN + 10% formic acid (FA) were added to fluroxypyr-meptyl containing samples and the mixtures were shaken vigorously by a vortex mixer for 10 min; (4) one Supel QuE Citrate Extraction Tube 55,227-U (Sigma–Aldrich, Taufkirchen, Germany) was added to abamectin containing samples/1 g sodium chloride and 4 g magnesium sulfate anhydrous were added to fluroxypyr-meptyl containing samples and centrifuge tubes were shaken immediately for 1 min; (5) centrifuge tubes were shaken vigorously by a vortex mixer for 10 min; (6) centrifuge tubes were centrifuged for 5 min at 3000 g and 10 °C; (7) 7 ml of the supernatant were transferred in 15 ml centrifuge tubes and ACN was evaporated with a nitrogen evaporator EVA 1 Vis (VLM GmbH, Bielefeld, Germany). The dry residues were redissolved in 0.25 ml of mobile phase (initial conditions) and transferred in autosampler vials for LC–MS/MS analysis.

### LC–MS/MS analysis

Abamectin is a mixture that contains ≥ 80% avermectin B1a and ≤ 20% avermectin B1b. Preliminary measurements showed that the avermectin B1b signals after sample preparation were below the limit of quantification. Therefore, all signals relate to avermectin B1a only.

Chromatographic separation was performed on an Agilent 1260 series liquid chromatography system (Agilent Technologies, Heilbronn, Germany) equipped with a reversed-phase Kinetex EVO C18 column (100 × 4.6 mm, 2.6 particle size, Phenomenex, Aschaffenburg, Germany) in combination with a guard column (Security Guard™ ULTRA, Phenomenex, Aschaffenburg, Germany). The liquid chromatography system consists of a binary pump system (G1312B), degasser (G4225A), autosampler with thermostat (G1367E HiP ALS + G1330B), column oven (G1316A TCC) and an Instant Pilot controller (G4208A). The injection volume was set to 2 µL and the flow rate was set to 0.5 mL/min for avermectin B1a method and 0.3 mL/min for fluroxypyr-meptyl method. The column oven was maintained at 30 °C. Gradient conditions for avermectin B1a analysis and for fluroxypyr-meptyl analysis can be found in the supplementary material (Table S1-2).

Analysis was carried out with an AB Sciex 6500 QTRAP system (Applied Biosystems, Toronto, Canada) equipped with an ESI source with positive and negative ionisation (positive: avermectin B1a and fluroxypyr-meptyl; negative: fluroxypyr) and multiple-reaction-monitoring mode. The operation parameters were as follows: ionspray voltage (IS) 5500 V, entrance potential (EP) 10 V, collision gas (CAD) medium, curtain gas (CUR) 40 psi, temperature of ion source (TEM) 400 °C, nebulising gas/ ion source gas 1 (GS1) 20 psi and drying gas/ ion source gas 2 (GS2) 50 psi. Parent ions were isolated and fragmented for avermectin B1a or fluroxypyr-meptyl and fluroxypyr. For each m/z transition, declustering potential (DP), collision energy (CE) and collision cell exit potential (CXP) were optimised to obtain the maximum intensities. Scheduled MRM detection window was set to 60 s and target scan time was set to 2.3251 s for avermectin B1a method and 1.1050 s for fluroxypyr-meptyl method. Parameters of detection are displayed in Table 2. Two m/z transitions with the highest intensity were obtained for each analyte, using the ion ratio as confirmatory parameter. Each sample was injected twice. Analyst Software was used for the LC–MS/MS system control and MultiQuant Software was used for data analysis.

**Table 2:** Parameters of detection

Analyte	Parent ion (m/z)	Product ions (m/z)	Retention time (min)	DP (V)	CE (V)	CXP (V)
Avermectin B1a	890.405	305.100	4:43	56	35	22
		567.300		56	19	40
		95.000		56	101	14
Fluroxypyr-meptyl	367.043	255.000	10:51	10	15	24
		209.000		10	31	14
		181.000		10	45	14
Fluroxypyr	252.781	194.700	3:22	− 10	− 14	− 21
		232.800		− 10	− 10	− 13
		188.800		− 10	− 20	− 17

### Statistical analysis

Graphical visualisation of the data was performed using Graphpad Prism software version 9.0.0 and statistical analysis was performed using R software version 4.0.3 (R Core Team 2020). For cell viability, fluorescence anisotropy and Pgp ATPase assay data a linear mixed-effects ANOVA (*α* = 0.05) described by Pinheiro and Bates (2000) followed by a post hoc Dunnett test of multiple comparisons of treatment groups vs. the control was used for statistical analysis. Calculations were performed using the R-packages nlme (Pinheiro et al. 2020) and multcomp (Hothorn et al. 2008).

For the transport studies a repeated measures linear mixed-effects ANOVA (*α* = 0.05) described by Pinheiro and Bates (2000) followed by a post hoc comparison with holm adjustment of the estimated curves of the combinations and products with the curve of the respective reference compound (active substance) was performed. Calculations were performed with the R-packages nlme (Pinheiro et al. 2020) and phia (De Rosario-Martinez et al. 2015).

## Results

### Cell viability

**Fig. 2:**
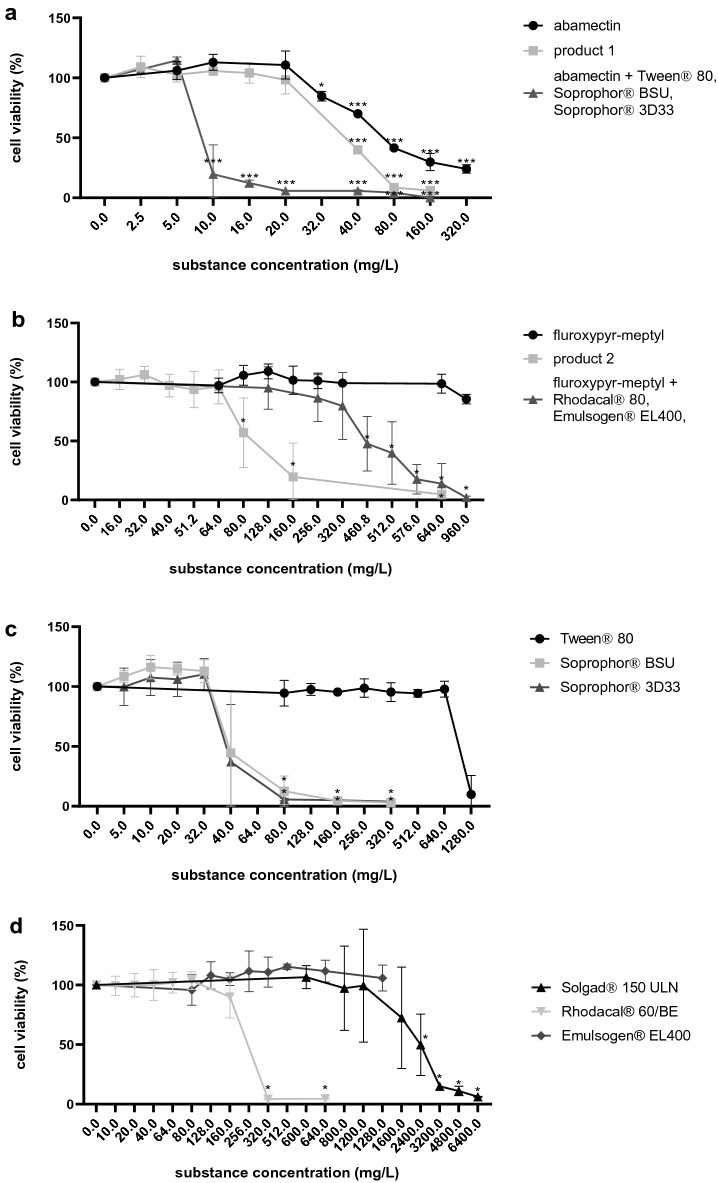
Results of the WST-1 cytotoxicity assay in Caco-2 cells after 24 h exposure to increasing concentrations of **a** abamectin, product 1 and the respective mixture of abamectin and the investigated co-formulants in the same ration as in product 1, **b** fluroxypyr-meptyl, product 2 and the respective mixtures of fluroxypyr-meptyl and the investigated co-formulants in the same ratio as in the product 2, **c** the investigated co-formulants of product 1 and **d** the investigated co-formulants of product 2. Results are shown as percentage of the viability of the solvent control containing 0.4% DMSO. Concentrations of the mixtures and the PPPs refer to the concentration of the contained active ingredient. Mean values ± SD of *n* = 3 biological replicates, each performed with six technical replicates. Statistical analysis was done by a linear mixed-effects ANOVA (*α* = 0.05) followed by a post hoc Dunnett test (*α* = 0.05). Statistical significance compared to solvent control is indicated by asterisks (*)

Results of WST-1 assays using Caco-2 cells and 24 h of incubation are shown in Fig. 2. Abamectin exhibited significant cytotoxic effects at 32 mg/L (about 85% viability). Product 1 exhibited significant and higher cytotoxic effects at 40 mg/L (about 40% viability) compared to abamectin. The respective mixture of the active substance and the surface active co-formulants (Tween^®^ 80, Soprophor^®^ BSU and Soprophor^®^ 3D33) in the same ratio as in the product exhibited already at 10 mg/L significant cytotoxic effects (about 20% viability) and was consequently most cytotoxic.

The second active ingredient fluroxypyr-meptyl did not show significant cytotoxic effects over the tested concentration range ($\le$ 960 mg/L). The respective mixture of the active substance and the surface active co-formulants (Rhodacal^®^ 60/BE and Emulsogen^®^ EL400) in the same ratio as in the product exhibited cytotoxic effects at 460.8 mg/L (about 50% viability). Product 2 exhibited cytotoxic effects at lower concentrations compared to the mixture (about 60% viability at 80 mg/L).

Tween^®^ 80 and Emulsogen^®^ EL400 were not cytotoxic in the tested concentration range ($\le$ 1280 mg/L). Soprophor^®^ BSU and Soprophor^®^ 3D33 exhibited significant cytotoxic effects at 80 mg/L, Rhodacal^®^60/BE at 320 mg/L and Solgad^®^ 150 ULN at 2400 mg/L.

Similar results were obtained with the NRU assay (a graphical visualisation can be found in Fig. S1 in the supplementary material).

### Concentration-additivity modelling

**Fig. 3:**
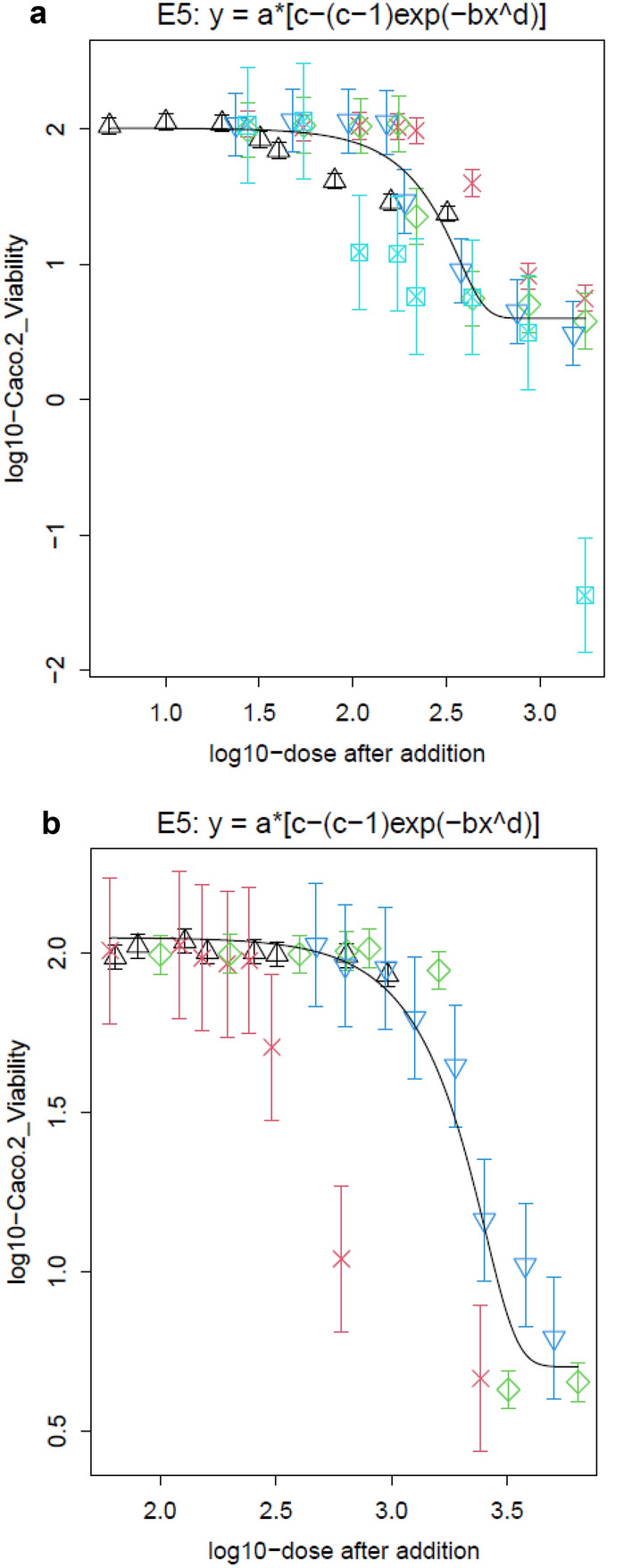
Concentration–response modelling of the cytotoxicity in Caco-2 cells of **a** abamectin (black upward triangle), Soprophor^®^ BSU (dark blue downward triangle), Soprophor^®^ 3D33 (green diamond), product 1 (red cross), combination of abamectin and respective co-formulants (light blue cross-square) and **b** fluroxypyr-meptyl (black upward triangle), Rhodacal^**®**^ 60/BE (green diamond), Solgad^®^ 150 ULN (dark blue downward triangle) and product 2 (red cross). The black lines are representing the dose–response curves of the mixtures under the assumption of dose addition of the single substances. Modelling was performed using the PROAST software ver. 70 (color figure online)

Abamectin was the reference substance for product 1. The relative potency factor (RPF) of Soprophor^®^ 3D33 was determined to be 4.895 and the RPF of Soprophor^®^ BSU was 5.678. We observe that the cytotoxicity dose–response data of the mixture of abamectin and the investigated co-formulants (light blue cross-square) is located left of the modelled dose–response curve representing dose addition. This indicates an effect which is more than additive. Product 1 (red cross) is located to the right of the curve indicating an effect which is less than additive (see Fig. 3a).

For the second comparison fluroxypyr-meptyl is the reference substance; Rhodacal^®^ 60/BE had a RPF of 9.796 and Solgad^®^ 150 ULN had a RPF of 0.6797. Since all co-formulants of product 2 are investigated, the PPP corresponds with the mixture of fluroxypyr-meptyl and the co-formulants. We see that the dose–response data of the product is situated to the left of the dose–response curve. This indicates a more than additive effect of the mixture (see Fig. 3b).

### Fluorescence anisotropy

Anisotropy values (*r* values) for untreated Caco-2 cells labelled with DPH were about 0.150.

**Fig. 4:**
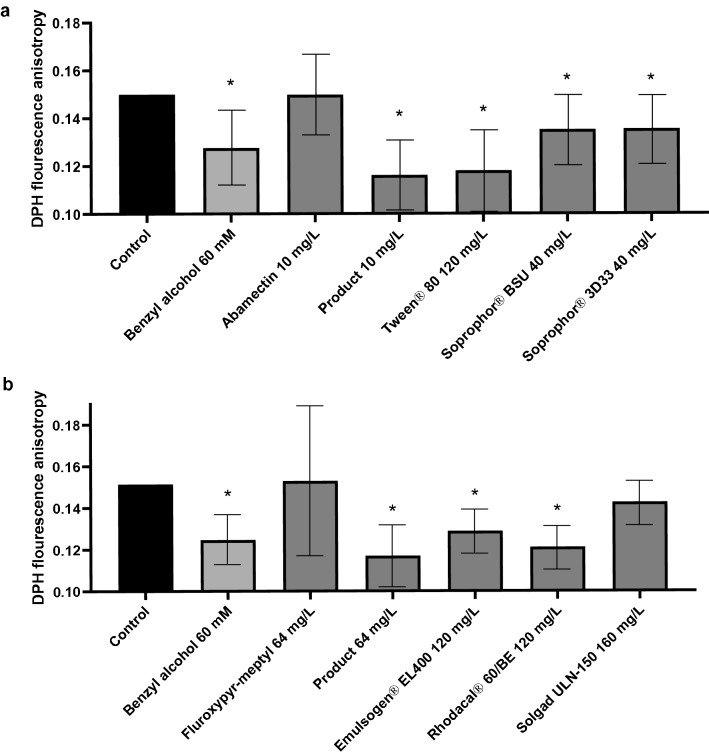
Alteration in Caco-2 cell membrane fluidity assessed using DPH fluorescence anisotropy after treatment with **a** abamectin, product 1, Tween^®^ 80, Soprophor^®^ BSU and Soprophor^®^ 3D33 and **b** fluroxypyr-meptyl, product 2, Rhodacal^®^ 60/BE, Emulsogen^®^ EL 400 and Solgad^®^ 150 ULN. Benzyl alcohol 60 mM served as a positive control. Data are presented as mean values of *n* = 3 biological replicates, each performed with two technical replicates. Error bars indicate confidence intervals obtained from statistical analysis using a two-sided post hoc Dunnett test (*α* = 0.05), with a preceding linear mixed-effects ANOVA test (*α* = 0.05). Statistical significance compared to untreated control is indicated by asterisks (*)

Results of compounds of product 1 are presented in Fig. 4a and of product 2 in Fig. 4b. All investigated surface active co-formulants showed a statistically significant decrease in DPH fluorescence anisotropy. 120 mg/L of Tween^®^ 80 and 120 mg/L of Emulsogen^®^ EL 400, both containing POE, caused a decrease in fluorescence anisotropy, resulting in mean *r* values of 0.135 and 0.139 respectively. Furthermore, 40 mg/L of the POE containing co-formulants Soprophor^®^ BSU and Soprophor^®^ 3D33 exhibited both a mean *r* value of 0.135. 120 mg/L of the anionic surfactant Rhodacal^®^ 60/BE decreased the fluorescence anisotropy significantly compared to the untreated control at a mean *r* value of 0.121.

10 mg/L of the active ingredient abamectin, 64 mg/L of the active ingredient fluroxypyr-meptyl as well as 160 mg/L of the solvent Solgad^®^ 150 ULN did not show a statistically significant impact on the fluorescence anisotropy. However, product 1 (containing 10 mg/L abamectin) and product 2 (containing 64 mg/L fluroxypyr-meptyl) exhibited significantly decreased *r* values of 0.116 and 0.117, respectively. In conclusion, the products showed the highest impact on the DPH fluorescence anisotropy.

Since all co-formulants of product 2 are examined here, the increased membrane fluidity caused by the product can be attributed to the surface active co-formulants. Product 1 contains additional, not investigated co-formulants. Since the membrane fluidity is still increased compared to the untreated control, the other co-formulants do not seem to compensate this effect.

### Pgp ATPase assay

**Fig. 5:**
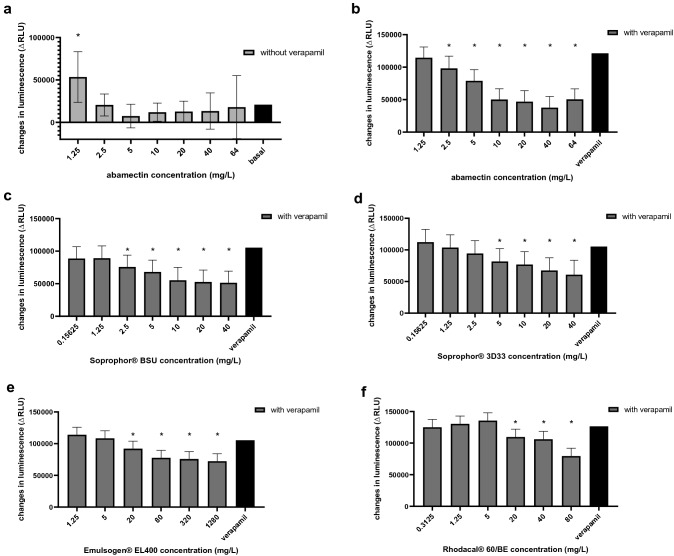
Pgp ATPase activity presented as changes in luminescence ΔRLU of (a) abamectin compared to untreated samples (basal) and (b–f) abamectin, Soprophor^®^ BSU, Soprophor^®^ 3D33, Emulsogen^®^ EL 400 and Rhodacal^®^ 60/BE after stimulation with verapamil compared to verapamil control. All measurements were corrected by subtraction of Na_3_VO_4_-treated signals (non Pgp ATPase activity). Data are presented as mean values of *n* = 4 independent experiments. Error bars indicate confidence intervals obtained from statistical analysis using an either two-sided (error bars in both direction) or one-sided (error bars in one direction) post hoc Dunnett test (*α* = 0.05), with a preceding linear mixed-effects ANOVA test (*α* = 0.05). Statistical significance compared to untreated control/verapamil control is defined by asterisks (*)

As shown in Fig. 5a, abamectin statistical significantly stimulated Pgp ATPase activity only at the lowest concentration of 1.25 mg/L by approximately 250% compared to the untreated control. Fluroxypyr-meptyl did not show a significant impact on the ATPase activity.

At higher concentrations (2.5–64 mg/L) abamectin inhibited in a concentration-dependent manner the verapamil-stimulated Pgp ATPase activity with a maximum decreased ΔRLU value of 30% compared to the verapamil control (Fig. 5b).

All investigated surfactants, except for Tween^®^ 80, showed inhibition of verapamil-stimulated Pgp ATPase activity in a concentration-dependent manner (Fig. 5c–f). Soprophor^®^ BSU and Soprophor^®^ 3D33 exhibited statistically significant decreased ΔRLU values at lowest concentrations of 2.5 mg/L and 5 mg/L, respectively. Emulsogen^®^ EL 400 and Rhodacal^®^ 60/BE decreased ΔRLU values at lowest concentrations of 20 mg/L. Furthermore, Solgad^®^ 150 ULN did not show significant inhibition of verapamil-stimulated Pgp ATPase activity.

In summary, four of five investigated surfactants are suggested to cause higher bioavailability of Pgp substrates (i.e. abamectin at low concentrations) due to Pgp ATPase inhibition. A graphical visualisation of the fluorescence anisotropy data for fluroxypyr-meptyl, Tween^®^ 80 and Solgad^®^ 150 ULN can be found in the supplementary material (Fig. S4).

### Transport studies

**Fig. 6:**
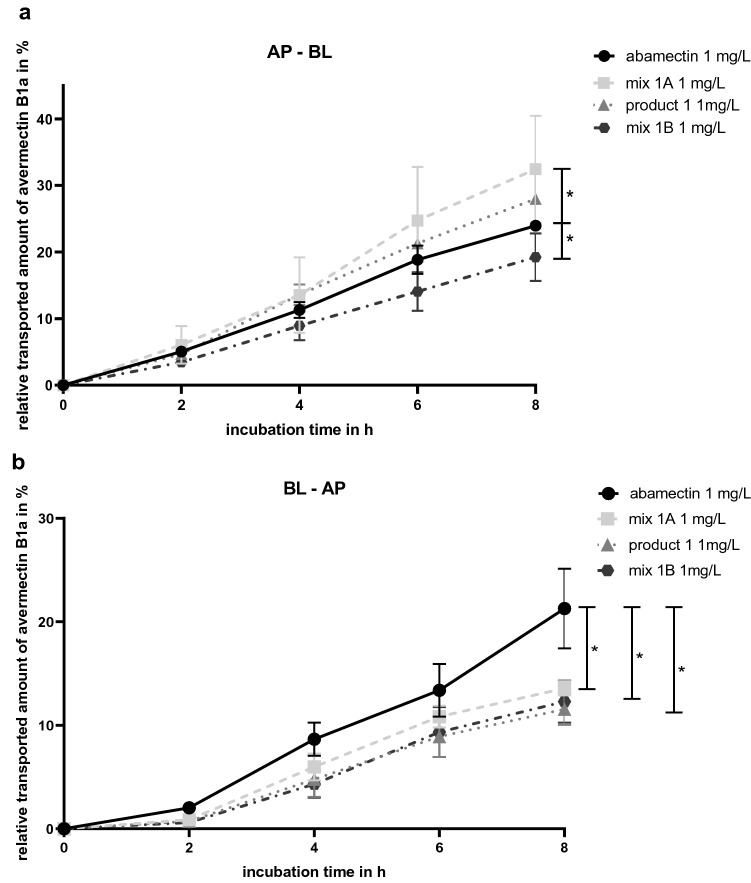
Results of transport studies presented as cumulative transported amounts of avermectin B1a in **a** AP–BL direction and **b** BL–AP direction. Transport studies in both directions were carried out with 1 mg/L abamectin, mix 1A (1 mg/L abamectin + 4.2 mg/L Tween^®^ 80, 1 mg/L Soprophor^®^ BSU, 1 mg/L Soprophor^®^ 3D33), product 1 (containing 1 mg/L abamectin) and mix 1B (1 mg/L abamectin + 80 mg/L Tween^®^ 80, 40 mg/L Soprophor^®^ BSU, 40 mg/L Soprophor^®^ 3D33). All concentrations were related to the measured start concentrations of the solutions and are given as relative values in percent. Data are presented as mean values ± SD of *n* = 3 independent experiments. Statistical analysis was conducted by a linear mixed-effects ANOVA (*α* = 0.05) followed by a post hoc comparison with holm adjustment of the estimated curves of mix 1A, product 1 and mix 1B with the curve of the active ingredient abamectin. Statistical significance at *α* = 0.05 level compared to reference compound is indicated by asterisks (*)

The relative transported amount of avermectin B1a in % over an incubation time of 8 h in both directions is shown in Fig. 6. In AP–BL direction, over an incubation time of 8 h with mix 1A significantly more avermectin B1a was absorbed compared to the transport studies with 1 mg/L of abamectin. After AP incubation with product 1 containing 1 mg/L abamectin, the same, but no significant trend was observed. In contrast, over 8 h of incubation with mix 1B, containing higher concentrations of the surfactants, less avermectin B1a was absorbed (Fig. 6a). In BL–AP direction, transport studies with all combinations (mix 1A, product 1 and mix 1B) resulted in significantly lower secretion of avermectin B1a, compared to those of abamectin alone (see Fig. 6b).

**Fig. 7:**
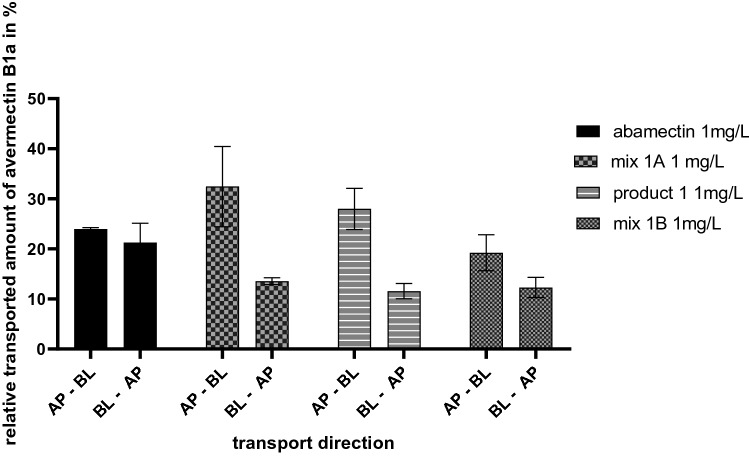
Comparison of the cumulative transported amount of avermectin B1a in % after 8 h of incubation in AP–BL and BL–AP direction carried out with 1 mg/L abamectin, mix 1A (1 mg/L abamectin + 4.2 mg/L Tween^®^ 80, 1 mg/L Soprophor^®^ BSU, 1 mg/L Soprophor^®^ 3D33), product 1 (containing 1 mg/L abamectin) and mix 1B (1 mg/L abamectin + 80 mg/L Tween^®^ 80, 40 mg/L Soprophor^®^ BSU, 40 mg/L Soprophor^®^ 3D33). Data are presented as mean values ± SD of *n* = 3 independent experiments

When comparing the AP–BL and BL–AP transport studies after 8 h of incubation, it was found that the studies with abamectin resulted in almost the same relative amounts of avermectin B1a in the BL compartment with 23.96% ± 0.29% and in the AP compartment with 21.27% ± 3.85% (see Fig. 7). Consequently, the BL–AP versus AP–BL ratio (efflux ratio) was close to 1 with a value of 0.888. Contrary, incubation with all combinations (mix 1A, product 1 and mix 1B) resulted in significantly higher amounts of avermectin B1a in the BL compartment compared to the AP compartment, suggesting higher absorption than secretion. The lowest efflux ratio was found for product 1 with 0.413, resulted by 27.99% ± 4.11% transported avermectin B1a amount in AP–BL direction and 11.56 ± 1.52% in BL–AP direction. Nearly the same efflux ratio was observed for mix 1A with 0.418, corresponding to 32.45% ± 0.29% of avermectin B1a in AP–BL direction and 13.57% ± 0.70% in BL–AP direction. For mix 1B in AP–BL direction 19.23% ± 3.59% and in BL–AP direction 12.29% ± 2.04% avermectin B1a was measured, leading to an efflux ratio of 0.636.

**Fig. 8:**
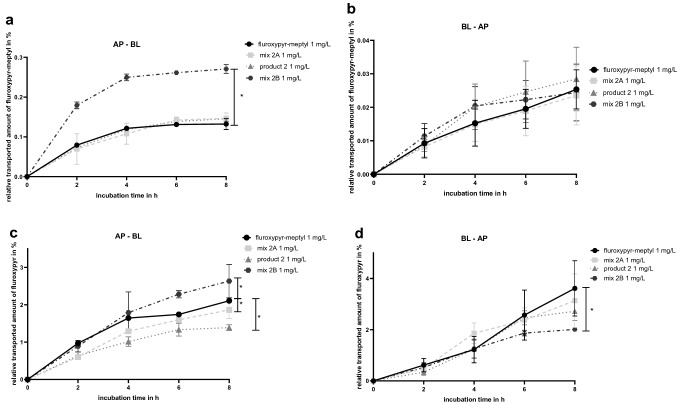
Results of transport studies presented as cumulative transported amounts of fluroxypyr-meptyl in **a** AP–BL direction and **b** BL–AP direction and fluroxypyr in **c** AP–BL direction and **d** BL–AP direction. Transport studies in both directions were carried out with 1 mg/L fluroxypyr-meptyl, mix 2A (1 mg/L fluroxypyr-meptyl + 0.1 mg/L Rhodacal^®^ 60/BE, 0.2 mg/L Emulsogen^®^ EL400), product 2 (containing 1 mg/L fluroxypyr-meptyl) and mix 2B (1 mg/L abamectin + 120 mg/L Rhodacal^®^ 60/BE, 120 mg/L Emulsogen^®^ EL400, 160 mg/L Solgad^®^ 150 ULN). All concentrations were related to the measured start concentrations of the solutions and are given as relative values in percent. Data are presented as mean values ± SD of *n* = 2 independent experiments for AP–BL direction and *n* = 3 independent experiments for BL–AP direction. Statistical analysis was done by a linear mixed-effects ANOVA (*α* = 0.05) followed by a post hoc comparison with holm adjustment of the estimated curves of mix 2A, product 2 and mix 2B with the curve of the active ingredient fluroxypyr-meptyl. Statistical significance at *α* = 0.05 level compared to reference compound is indicated by asterisks (*)

The relative transported amount of fluroxypyr-meptyl and its metabolite fluroxypyr over an incubation time of 8 h in both directions is shown in Fig. 8. In AP–BL direction, incubation with mix 2B resulted in a significantly higher absorption of fluroxypyr-meptyl and fluroxypyr compared to the transport studies with 1 mg/L of fluroxypyr-meptyl. Mix 2A and product 2, both containing lower concentrations of the surfactants, did not show a significant difference of the transported fluroxypyr-meptyl amount compared to transport studies incubated with fluroxypyr-meptyl alone (Fig. 8a). In contrast, after incubation with mix 2A and product 2 in AP–BL direction a higher relative amount of the metabolite fluroxypyr was measured than in fluroxypyr-meptyl transport studies (Fig. 8c).

Furthermore, in BL–AP direction, incubation with all combinations and the active ingredient individually resulted in comparable transported fluroxypyr-meptyl amounts (Fig. 8b). Similar results were found for the metabolite, except for the results after incubation with mix 2B. Significantly less fluroxypyr was secreted over 8 h of incubation with mix 2B compared to transport studies with the active ingredient (Fig. 8d).

**Fig. 9:**
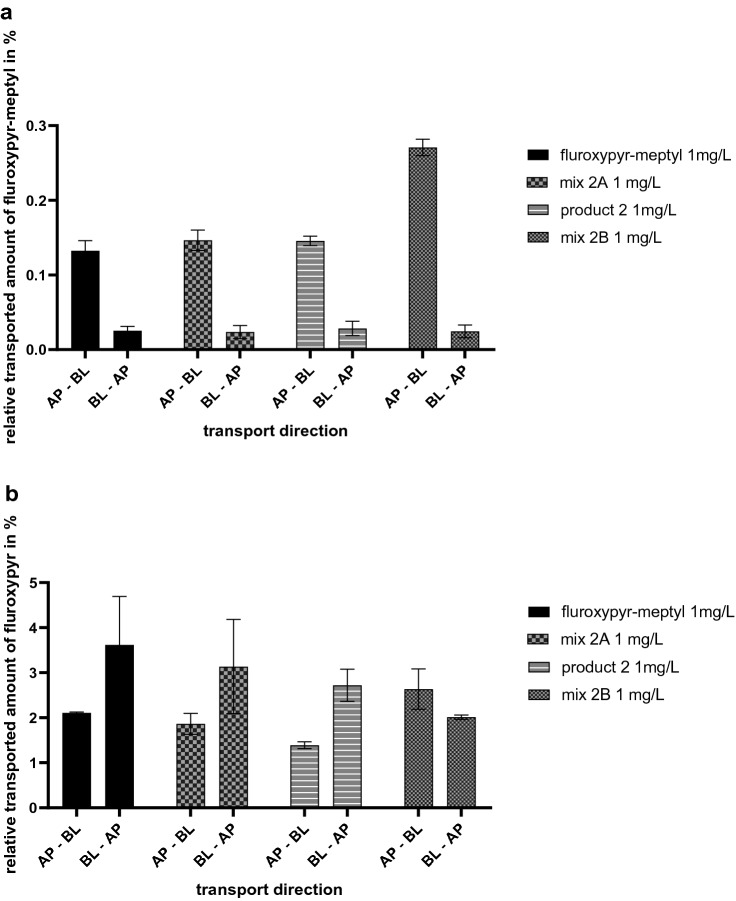
Comparison of the cumulative transported amount of **a** fluroxypyr-meptyl and **b** fluroxypyr in % after 8 h of incubation in AP–BL and BL–AP direction carried out with 1 mg/L fluroxypyr-meptyl, mix 2A (1 mg/L fluroxypyr-meptyl + 0.1 mg/L Rhodacal^®^ 60/BE, 0.2 mg/L Emulsogen^®^ EL400), product 2 (containing 1 mg/L fluroxypyr-meptyl) and mix 2B (1 mg/L abamectin + 120 mg/L Rhodacal^®^ 60/BE, 120 mg/L Emulsogen^®^ EL400, 160 mg/L Solgad^®^ 150 ULN). Data are presented as mean values ± SD of *n* = 2 independent experiments for AP–BL direction and *n* = 3 independent experiments for BL–AP direction

When comparing the AP–BL and BL–AP direction after 8 h, incubation with all treatments resulted in higher amounts of fluroxypyr-meptyl in the BL compartment compared to the AP compartment. The lowest efflux ratio of 0.089 was found for mix 2B with 0.271% ± 0.011% of fluroxypyr-meptyl in AP–BL direction and 0.024% ± 0.008% in BL–AP direction. 0.132% ± 0.014% of fluroxypyr-meptyl was obtained after incubation with the active ingredient individually in AP–BL direction and 0.025% ± 0.006% in BL–AP direction, resulting in an efflux ratio of 0.189. For mix 2A and product 2 similar efflux ratios of 0.164 and 0.193 were obtained, respectively (Fig. 9a).

Notably, comparing fluroxypyr amounts in AP–BL and BL–AP direction, incubation with the active ingredient individually, mix 2A and product 2 showed higher amounts of the metabolite in BL–AP direction than in AP–BL direction. 2.107% ± 0.017% of fluroxypyr was obtained after incubation with fluroxypyr-meptyl in AP–BL direction and 3.614% ± 1.079% in BL–AP direction, resulting in a flux of 1.715. For mix 2A and product 2 similar efflux ratios of 1.683 and 1.958 were found, respectively. Only mix 2B showed higher amounts of fluroxypyr in AP–BL direction (2.624% ± 0.447%) compared to BL–AP direction (2.011% ± 0.050%), resulting in an efflux ratio of 0.763 (Fig. 9b).

## Discussion

The aim of this work was to investigate mixture effects of surface active co-formulants and active substances in PPPs on the toxicokinetic level due to altered absorption and/or secretion. To address this issue, two PPPs were selected, containing several surface active co-formulants. Our results show that the surface active co-formulants investigated in this work exhibit toxicokinetic interactions on absorption and secretion level leading to an increased bioavailability of the respective active substances and in terms of mixture toxicity also, at least partially, to deviations from the assumed concentration-addition model. Depending on the concentration of the surfactants, Pgp ATPase inhibition as well as membrane fluidisation were detected as possible mechanistic explanations for most of the surfactants.

The importance of toxicokinetic interactions was already described in several systematic review articles, e.g. by Cedergreen (2014); Martin et al. (2021). Generally, these indicate synergistic effects to be rare. However, if they occur, they usually seem rooted in toxicokinetic interactions, especially metabolic ones (Martin et al. 2021). Befittingly, a toxicokinetic interaction with CYP enzymes that leads to a more than additive mixture effect on triglyceride accumulation in human liver cells has only been described recently (Lasch et al. 2021).

In this study, both products and combinations exhibited increased cytotoxic effects at lower concentrations than the active ingredients. Since some of the investigated co-formulants exhibited cytotoxic effects individually, concentration-additivity modelling was used to analyse potential synergistic effects due to toxicokinetic interactions.

More than additive mixture effects were found for the cytotoxicity of the combination of abamectin and the surfactants Tween^®^ 80, Soprophor^®^ BSU and Soprophor^®^ 3D33, contained in product 1. Previously, such a synergistic effect of the non-ionic surfactants Tween^®^ 80 and PEG6000 on cytotoxicity of insecticides, including abamectin was described by Li et al. (2015). However, the underlying mechanisms of interaction remained unresolved.

Our findings support the hypothesis that the surface active co-formulants increase absorption in relation to secretion, leading to increased BL concentrations of the active substances. Since the efflux ratio for product 1 was not noticeable lower compared to the combinations with the surfactants, the impact found in the transport studies can likely be attributed to the surface active co-formulants. The higher impact on secretion emphasises the involvement of active transporters in secretory direction, such as Pgp transporters (Aungst 1999; Troutman and Thakker 2003). These findings are in line with the Pgp ATPase assay results, showing Pgp inhibition caused by Soprophor^®^ BSU and Soprophor^®^ 3D33.

Furthermore, all of the three surfactants increased membrane fluidity, which partially explains the more than additive effect. Tween^®^ 80 has previously been described to cause increased membrane fluidity of Caco-2 cells below and above their respective critical micelle concentrations (Greulich 2003; Rege et al. 2002). However, this has not been described for the other two substances Soprophor^®^ BSU and Soprophor^®^ 3D33.

Surprisingly, mix 1A containing higher concentrations of Tween^®^ 80 (80 mg/L), Soprophor^®^ BSU (40 mg/L) and Soprophor^®^ 3D33 (40 mg/L) resulted in a lower absorbed relative amount of avermectin B1a in AP–BL transport studies. A possible explanation could be the formation of mixed micelles due to the high concentrations of the surfactants, leading to a micellar inclusion of the abamectin. This would result in a reduced freely transportable amount of abamectin. Notably, it has been described that the Pgp inhibition caused by surfactants decreases above the critical micelle concentration (Nerurkar et al. 1996).

A second more than additive mixture effect was found for the cytotoxicity of product 2 (combination of fluroxypyr-meptyl, Rhodacal^®^ 60/BE Emulsogen^®^ EL400 and Solgad^®^ 150 ULN). In summary, the more than additive mixture effect shown for product 2 seems to be due to solubilisation, since the efflux ratio in the transport studies for the product and the active ingredient were nearly the same. However, depending on the concentration of the surfactants and the active ingredients further toxicokinetic interactions are possible, as shown for mix 2B, which contains fluroxypyr-meptyl and higher concentrations of the surfactants than in the product.

The hypothesis that further toxicokinetic interaction is possible at higher concentrations was also supported by additional results. Both surfactants increased membrane fluidity and inhibited verapamil-stimulated Pgp ATPase activity at higher concentrations. The concentrations of the surfactants in mix 2A and product 2 (0.1 mg/L Rhodacal^®^ 60/BE and 0.2 mg/L Emulsogen^®^ EL400) are distinctly lower than the concentrations, that showed effects in the Pgp ATPase assays and in the fluorescence anisotropy measurements. Cremophor^®^ EL (polyoxyethylene 35 castor oil CAS No. same as Emulsogen^®^ EL400) has been described in previous studies to increase membrane fluidity in Caco-2 cells (Greulich 2003; Rege et al. 2002). Furthermore, our results for Emulsogen^®^ EL400 are in line with previous inhibition studies of rhodamine 123 transport in Caco-2 cells caused by Cremophor^®^ EL, suggesting a Pgp inhibition by these surfactants (Hugger et al. 2002; Rege et al. 2002).

Furthermore, mix 2B showed a reversal of the efflux ratio of the metabolite fluroxypyr, indicating a net absorption. This is a further indication of the involvement of active transporters, which are inhibited by the surfactants at higher concentrations. An interaction with Pgp transporters is possible. Other secretory transporters expressed in Caco-2 cells, such as multidrug resistance-associated proteins 2 (MRP2) could also be responsible, as previously described: MRP2 transporters may be inhibited by several surfactants such as Cremophor^®^ EL, Cremophor^®^ RH40 and Solutol^®^ HS15, PEG 2000 and pluronic block copolymers (Greulich 2003; Li et al. 2014).

Our findings as well as recent investigations imply that a more systematic consideration of toxicokinetic mixture effects is required, at least for selected examples. Nevertheless, since toxicokinetic interactions are highly related to the concentrations investigated, approaches are needed to consider concentration-dependent effects due to co-formulants in PPP risk assessment.

Implemented grouping approaches for mixture selection, such as the common assessment groups (CAG) and the adverse outcome pathway (AOP), are based on toxicodynamic characteristics, whereby toxicokinetic interactions are not addressed in similar detail. This is different in respective regulations related to pharmaceuticals (ICH 1994). The development of a grouping approach based on toxicokinetic properties could hence be a way forward (Braeuning and Marx-Stoelting 2021). Concomitantly, with regard to hazard assessment based on the CLP calculation method, it should be critically assessed under which conditions the assumption of additivity is appropriate and when one would have to consider over-additivity due to toxicokinetic interactions (Hernandez et al. 2013; Kurth et al. 2019). Previous studies have hence suggested a novel tiered test strategy that considers both, toxicodynamic as well as toxicokinetic interactions (Bloch et al. 2020). Grouping approaches for toxicokinetic properties could be a useful tool for amending such an approach. Specifically, surfactants were found to modulate membrane fluidity and interact with Pgp efflux transporters, altering active substance net uptake and secretion.

## Supplementary Information

Below is the link to the electronic supplementary material.

Supplementary file1 Table S1 Gradient conditions for avermectin B1a analysis. Table S2 Gradient conditions for fluroxypyr-meptyl analysis. Fig. S1 Results of the NRU cytotoxicity assay in Caco-2 cells after 24 h exposure to increasing concentrations of the investigated substances. Fig. S2 Results of FITC-dextran flux (Papp values) representing cell layer integrity in transport studies. Fig. S3 Results of transepithelial electrical resistance measurements (TEER values) representing cell layer integrity in transport studies. Fig. S4 Pgp ATPase activity presented as changes in luminescence ΔRLU of (A) fluroxypyr-meptyl compared to untreated samples (basal) and (B) Tween® 80 and Solgad® 150 ULN after stimulation with verapamil compared to verapamil control. (PDF 874 KB)

## Data Availability

Not applicable.

## Abbreviations

ΔRLU: Change in relative light units
ACC-1B: Accutase cell detachment solution
ACN: Acetonitrile
AOP: Adverse outcome pathway
AP: Apical
BL: Basolateral
CAD: Collision gas
CAG: Common assessment groups
CE: Collision energy
CUR: Curtain gas
CXP: Collision cell exit potential
DMEM: Dulbecco’s Modified Eagle Medium
DMSO: Dimethyl sulfoxide
DP: Declustering potential
DPH: 1,6-Diphenyl-1,3,5-hexatriene
EP: Entrance potential
FA: Formic acid
FCS: Fetal calf serum
FITC-Dextran: Fluorescein isothiocyanate-dextran
GS1: Nebulizing gas/ion source gas 1
GS2: Drying gas/ion source gas 2
IS: Ionspray voltage
MDR1: Multidrug resistance protein 1
MRP2: Multidrug resistance-associated proteins 2
NRU: Neutral red uptake
Papp: Apparent permeability coefficients
PBS: Phosphate-buffered saline
Pgp: P-glycoproteins
POE: Polyoxyethylene
PPP: Plant protection products
QuEChERS: Quick, Easy, Cheap, Effective, Rugged and Safe
WST-1: Water soluble tetrazolium
RPF: Relative potency factor
*r* value: Anisotropy value
SD: Standard deviation
TEER: Transepithelial electrical resistance
TEM: Temperature of ion source
THF: Tetrahydrofuran
