# Supplementary material for: Effects of co-formulants on the absorption and secretion of active substances in plant protection products in vitro
Source: Arch Toxicol. 2021 Aug 20;95(10):3205–21. doi: 10.1007/s00204-021-03140-x (PMC8448693; doi:10.1007/s00204-021-03140-x)
Supplement: Supplementary file 1 — Supplementary file1 Table S1 Gradient conditions for avermectin B1a analysis. Table S2 Gradient conditions for fluroxypyr-meptyl analysis. Fig. S1 Results of the NRU cytotoxicity assay in Caco-2 cells after 24 h exposure to increasing concentrations of the investigated substances. Fig. S2 Results of FITC-dextran flux (Papp values) representing cell layer integrity in transport studies. Fig. S3 Results of transepithelial electrical resistance measurements (TEER values) representing cell layer integrity in transport studies. Fig. S4 Pgp ATPase activity presented as changes in luminescence ΔRLU of (A) fluroxypyr-meptyl compared to untreated samples (basal) and (B) Tween® 80 and Solgad® 150 ULN after stimulation with verapamil compared to verapamil control. (PDF 874 KB) [file 204_2021_3140_MOESM1_ESM.pdf]

Toxicokinetic mixture effects of co-formulants and active substances in plant protection products *in vitro*

Karaca, Mawien[1,2], Fischer, Benjamin[1], Willenbockel, Christian Tobias[1], Tewes Tralau[1], Marx-Stoelting, Philip[1,2]*, Bloch, Denise[1]*

[1] German Federal Institute for Risk Assessment, Department of Pesticides Safety, Max-Dohrn-Straße 8-10, 10589 Berlin, Germany

[2] Technical University of Berlin, Institute for Chemistry, Straße des 17. Juni 115, 10623 Berlin, Germany

* Correspondence to: Marx-Stoelting, P. or Bloch, D.; German Federal Institute for Risk Assessment, Berlin 1589 Berlin, Germany; E-mail: Philip.Marx-Stoelting@bfr.bund.de or Denise.Bloch@bfr.bund.de

Bloch, D. and Marx-Stoelting, P. contributed equally to this paper

Supplementary Information

a

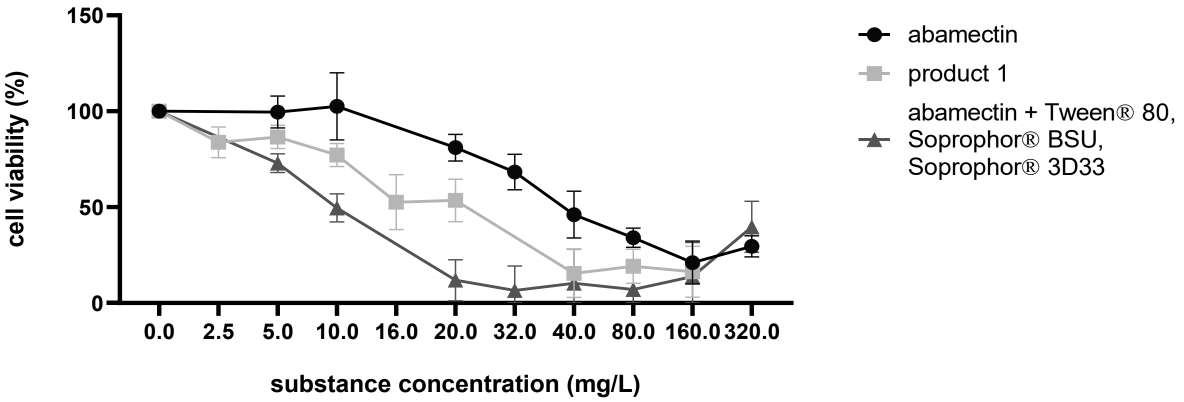

b

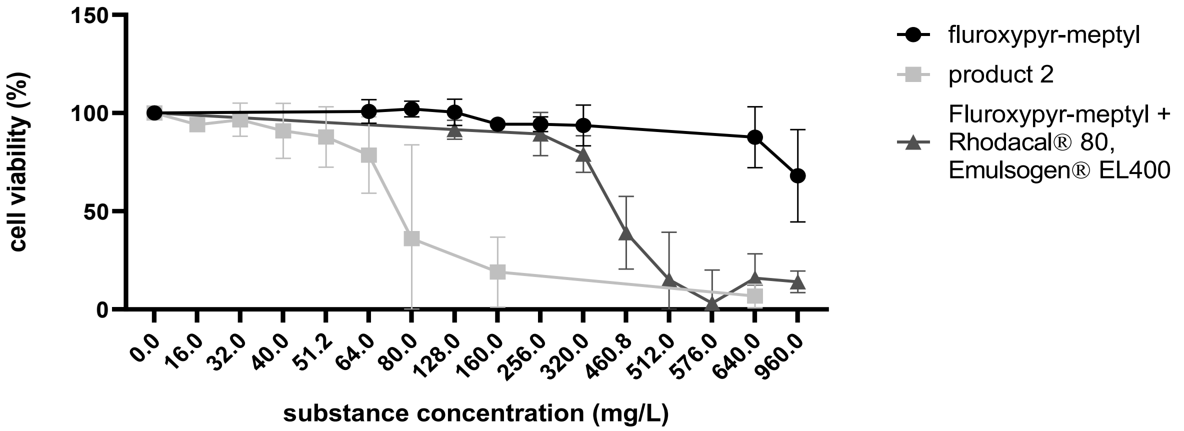

c

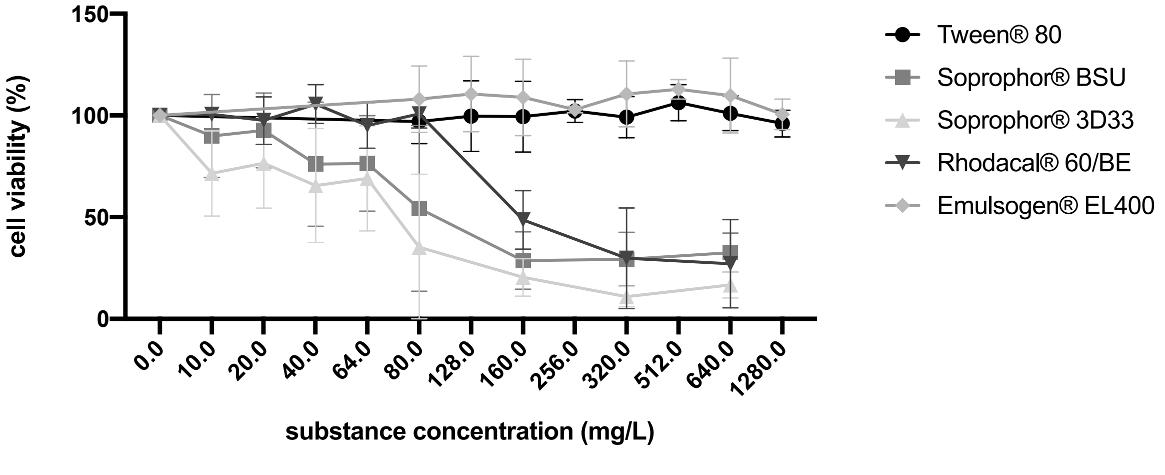

Fig. S1 Results of the NRU cytotoxicity assay in Caco-2 cells after 24 h exposure to increasing concentrations of the sinlge active ingredients and investigated co-formulants, their respective mixtures in the same ratio as in the PPPs and the PPPs. Results are shown as percentage of the viability of the solvent control containing 0.4% DMSO. Triton-X served as a positive control. Concentrations of the mixtures and the PPPs are related to the concentration of the contained active ingredient. Mean values ± SD of n = 3 biological replicates, each performed with six technical replicates, are given.

a

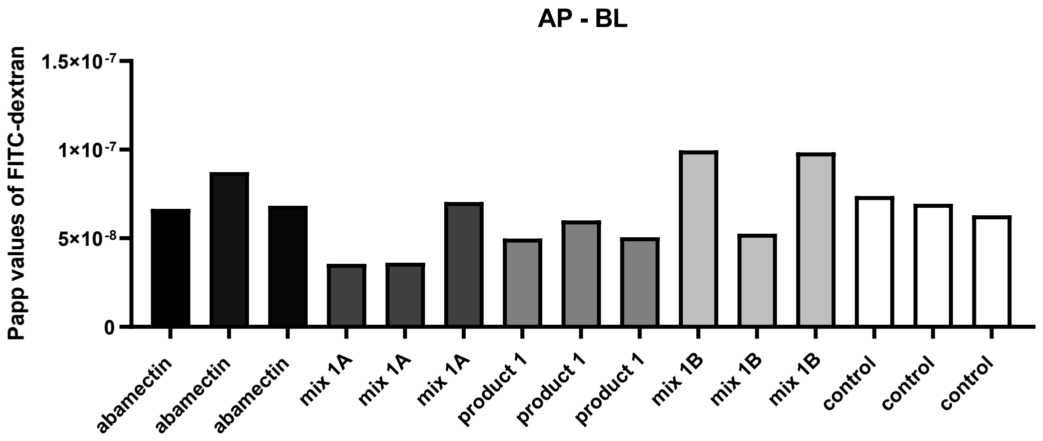

b

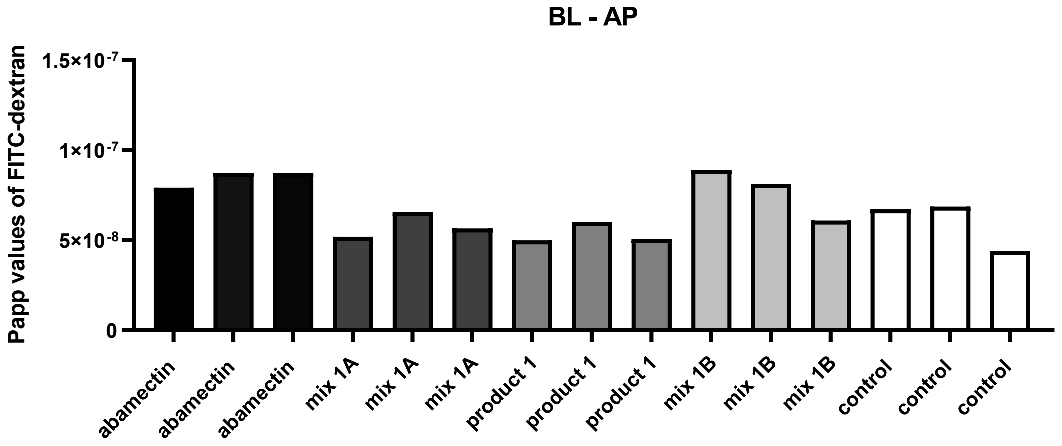

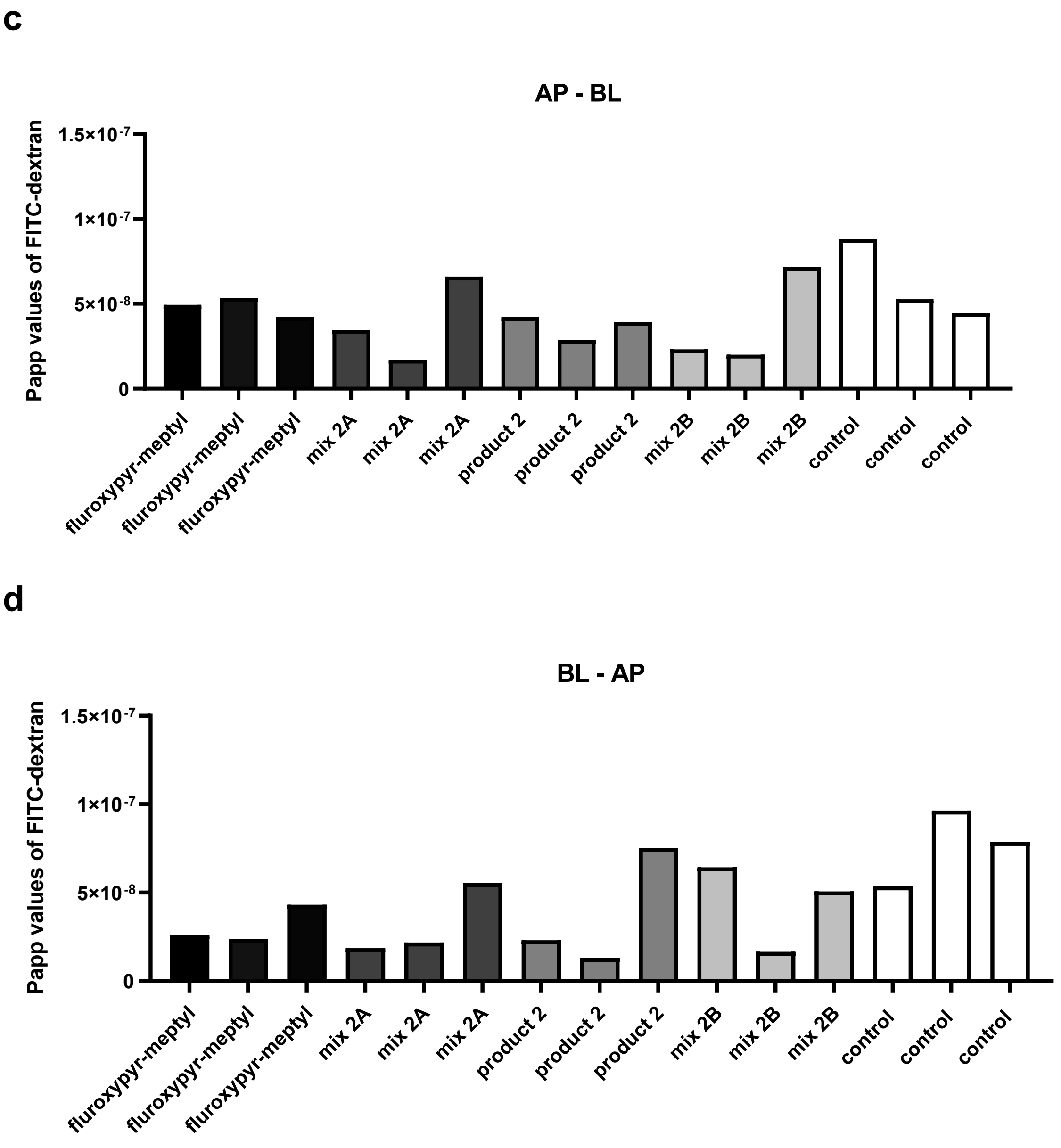

Fig. S2 Results of FITC-dextran flux (Papp values) representing cell layer integrity in transport studies of (a-b) 1 mg/L abamectin, mix 1A (1 mg/L abamectin + 4,2 mg/L Tween® 80, 1 mg/L Soprophor® BSU, 1 mg/L Soprophor® 3D33), product 1 (containing 1 mg/L abamectin) and mix 1B (1 mg/L abamectin + 80 mg/L Tween® 80, 40 mg/L Soprophor® BSU, 40 mg/L Soprophor® 3D33) and (b-c) 1 mg/L fluroxypyr-meptyl, mix 2A (1 mg/L fluroxypyr-meptyl + 0.1 mg/L Rhodacal® 60/BE, 0.2 mg/L Emulsogen® EL400), product 2 (containing 1 mg/L fluroxypyr-meptyl) and mix 2B (1 mg/L abamectin + 120 mg/L Rhodacal® 60/BE, 120 mg/L Emulsogen® EL400, 160 mg/L Solgad® 150 ULN).

a

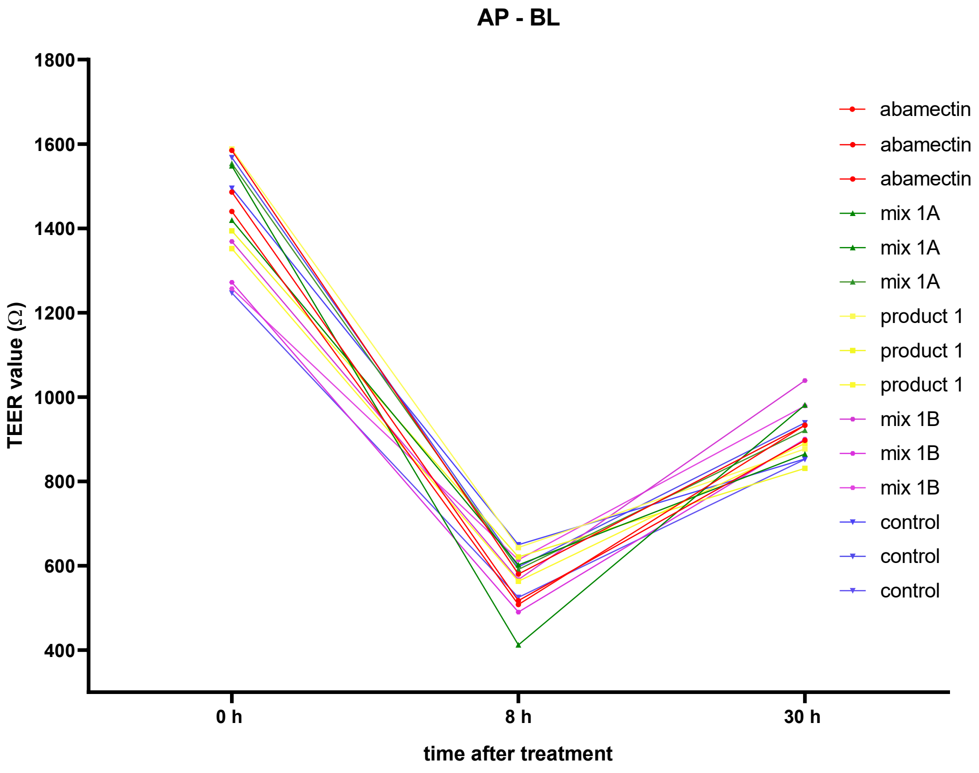
AP - BL
TEER value (Ω)
1800
1600
1400
1200
1000
800
600
400
0 h
8 h
30 h
time after treatment
abamectin
abamectin
abamectin
mix 1A
mix 1A
mix 1A
product 1
product 1
product 1
mix 1B
mix 1B
mix 1B
control
control
control

b

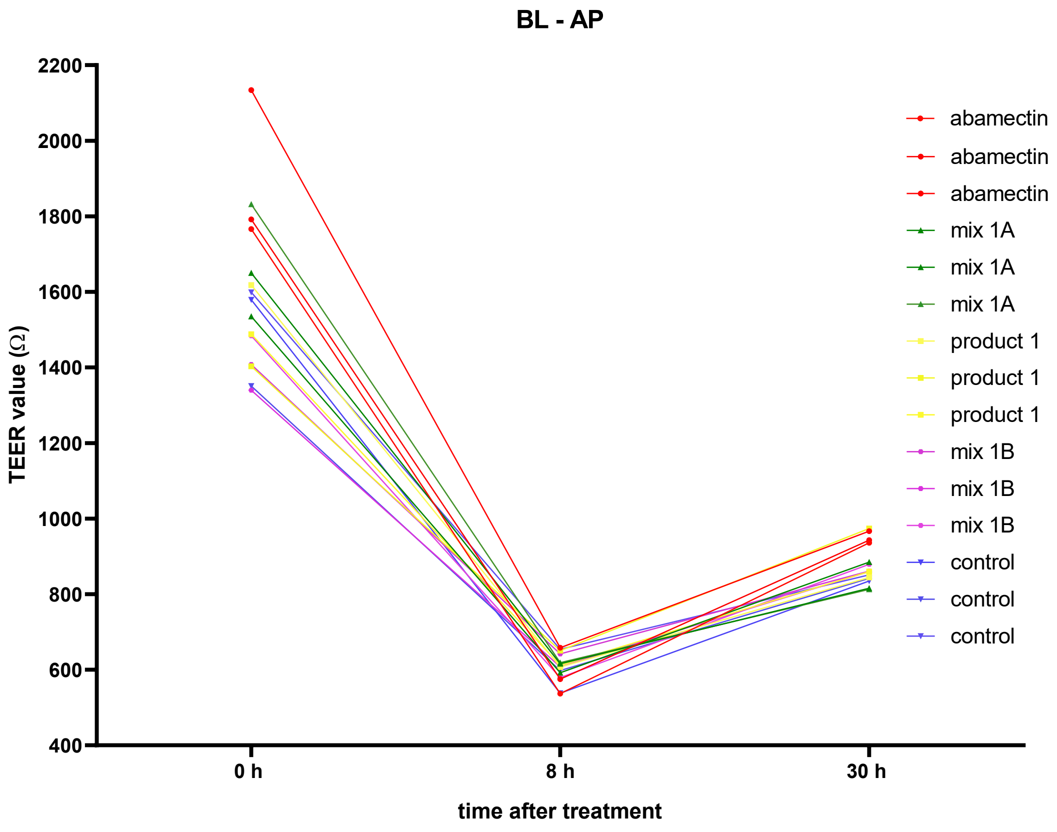
BL - AP
TEER value (Ω)
2200
2000
1800
1600
1400
1200
1000
800
600
400
0 h
8 h
30 h
time after treatment
abamectin
abamectin
abamectin
mix 1A
mix 1A
mix 1A
product 1
product 1
product 1
mix 1B
mix 1B
mix 1B
control
control
control

c

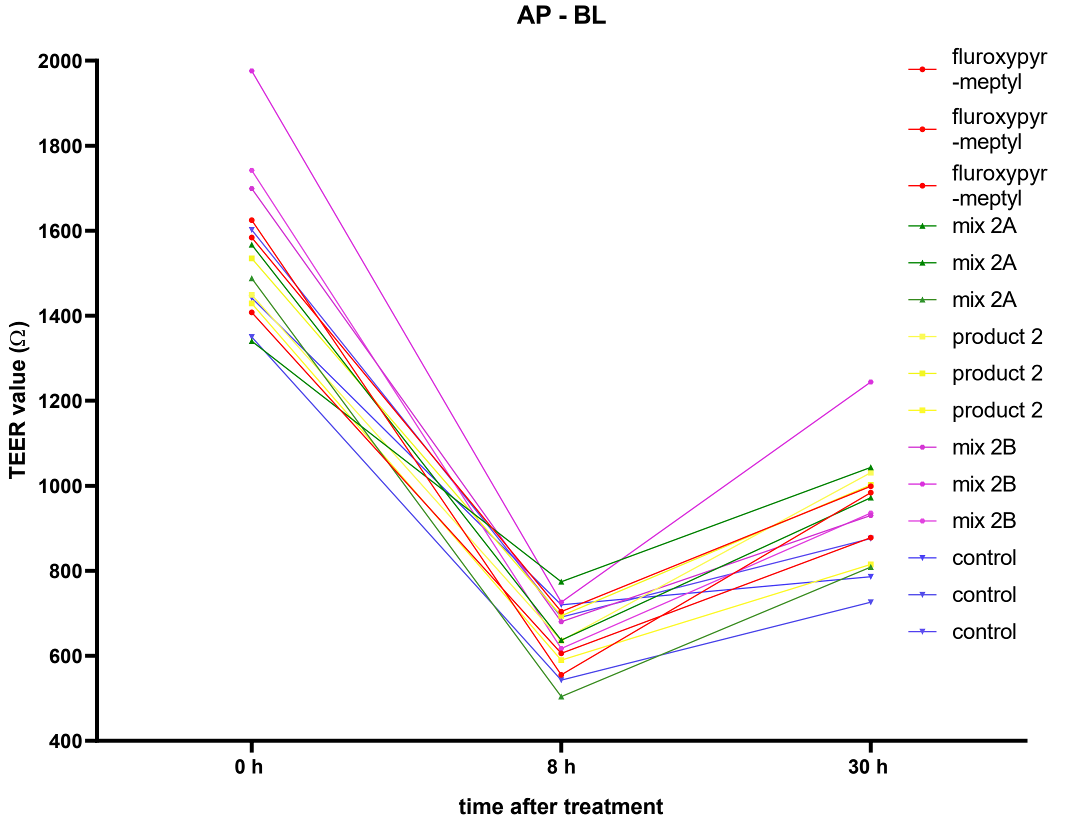
AP - BL
TEER value (Ω)
2000
1800
1600
1400
1200
1000
800
600
400
0 h
8 h
30 h
time after treatment
fluroxypyr -meptyl
fluroxypyr -meptyl
fluroxypyr -meptyl
mix 2A
mix 2A
mix 2A
product 2
product 2
product 2
mix 2B
mix 2B
mix 2B
control
control
control

d

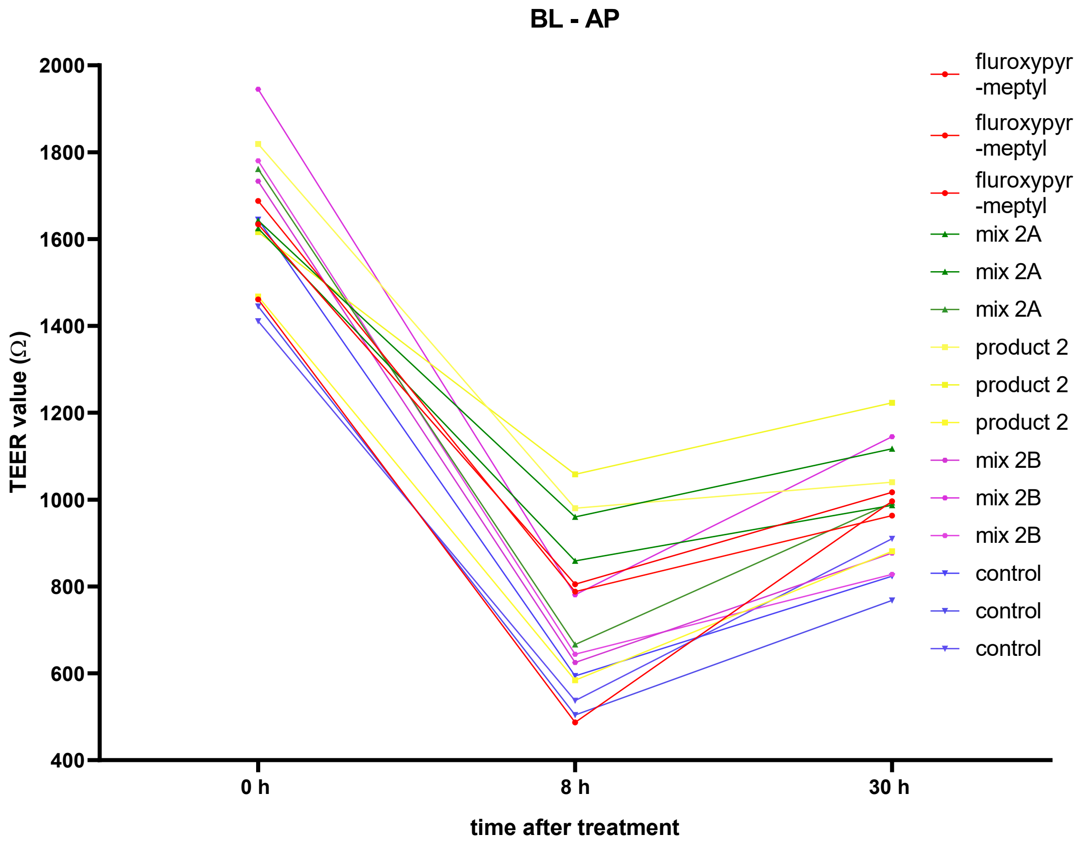
BL - AP
TEER value (Ω)
2000
1800
1600
1400
1200
1000
800
600
400
0 h
8 h
30 h
time after treatment
fluroxypyr -meptyl
fluroxypyr -meptyl
fluroxypyr -meptyl
mix 2A
mix 2A
mix 2A
product 2
product 2
product 2
mix 2B
mix 2B
mix 2B
control
control
control

Fig. S3 Results of transepithelial electrical resistance measurements (TEER values) representing cell layer integrity in transport studies of (a-b) 1 mg/L abamectin, mix 1 (1 mg/L abamectin + 4,2 mg/L Tween® 80, 1 mg/L Soprophor® BSU, 1 mg/L Soprophor® 3D33), product 1 (containing 1 mg/L abamectin) and mix 2 (1 mg/L abamectin + 80 mg/L Tween® 80, 40 mg/L Soprophor® BSU, 40 mg/L Soprophor® 3D33) and (c-d) 1 mg/L fluroxypyr-meptyl, mix 1 (1 mg/L fluroxypyr-meptyl + 0.1 mg/L Rhodacal® 60/BE, 0.2 mg/L Emulsogen® EL400), product 2 (containing 1 mg/L fluroxypyr-meptyl) and mix 2 (1 mg/L abamectin + 120 mg/L Rhodacal® 60/BE, 120 mg/L Emulsogen® EL400, 160 mg/L Solgad® 150 ULN).

a

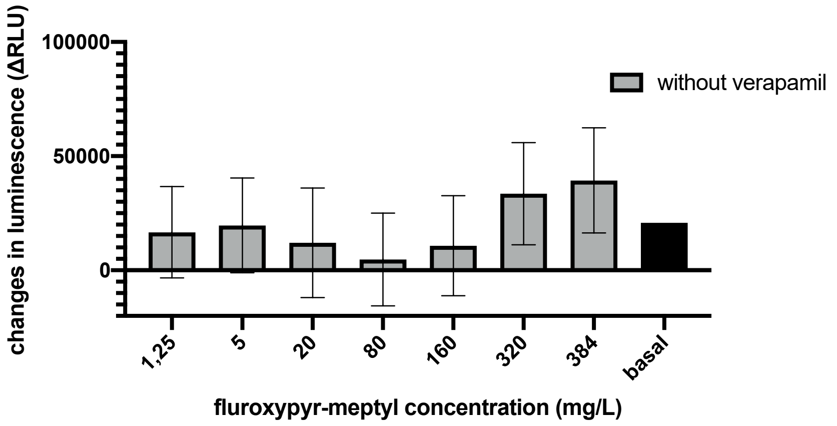

b

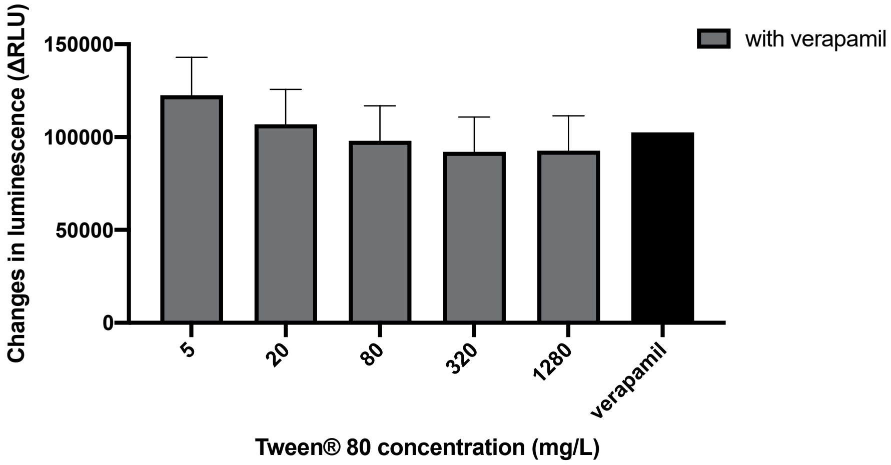

c

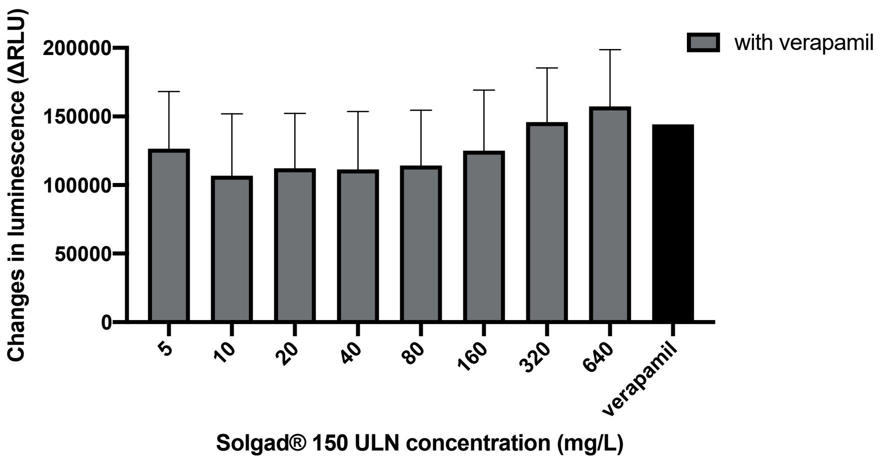

Fig. S4 Pgp ATPase activity presented as changes in luminescence ΔRLU of (a) fluroxypyr-meptyl compared to untreated samples (basal) and (b) Tween® 80 and (c) Solgad® 150 ULN after stimulation with verapamil compared to verapamil control. All measurements were corrected by subtraction of Na_3VO_4 treated signals (non Pgp ATPase activity). Data are presented as mean values of n = 4 independent experiments. Error bars indicate confidence intervals obtained from statistical analysis using an either two-sided (error bars in both direction) or one-sided (error bars in one direction) post-hoc Dunnett-test, with a preceding linear mixed-effects ANOVA test ($\alpha = 0.05$).

Table S1

Gradient conditions for avermectin B1a analysis

Time (min:sec)	% A (0.1% (v/v) formic acid in water)	% B (0.1% (v/v) formic acid in acetonitrile)
0:00	20	80
1:00	5	95
3:00	5	95
3:50	20	80
8:00	20	80

Table S2

Gradient conditions for fluroxypyr-meptyl analysis

Time (min:sec)	% A (0.1% (v/v) formic acid in water)	% B (0.1% (v/v) formic acid in acetonitrile)
0:00	30	70
2:00	30	70
4:00	10	90
10:00	10	90
10:05	30	70
13:00	30	70
